# A systematic literature review and meta-analysis of characterization of canine parvoviruses 2 prevalent in mainland China

**DOI:** 10.1186/s12985-020-01462-3

**Published:** 2020-12-11

**Authors:** Bo Dong, Gaoqiang Zhang, Jiajia Zhang, Junyu Bai, Weiming Lin

**Affiliations:** 1 College of Life Science of Longyan University, Longyan, 364012 China; 2 Fujian Provincial Key Laboratory for the Prevention and Control of Animal Infectious Diseases and Biotechnology, Longyan, 364012 China; 3 Longyan University Animal Hospital, Longyan, 364012 China; 4 Shenzhen An An Animal Hospital, Shenzhen, 518000 China

**Keywords:** CPV-2, Systematic review, Meta-analysis

## Abstract

**Background:**

Canine parvovirus 2 (CPV-2) is a pathogenic virus that infects dogs, causing a highly infectious disease. Monitoring CPV-2 spread is an important part of prevention; however, the prevalence and epidemiological characteristics of CPV-2 have not been systematically evaluated and analyzed in mainland China. Therefore, a systematic review and meta-analysis were performed to assess prevalence and epidemiological characteristics of CPV-2 in domestic dogs in mainland China.

**Methods:**

In this study, Chinese and English literature on CPV-2 epidemiology published between January 2006 and December 2019 was evaluated. Regarding meta-analysis, the random-effect model was employed by forest plot with 95% of confidence interval. The number of CPV-2 infections was identified and the pooled prevalence of infection, as well as the epidemiological characteristics, was calculated using meta-analysis.

**Results:**

A total of 39 studies (data from 137,844 dogs) met the evaluation criteria and were used in our study. The pooled prevalence of CPV-2 infection in mainland China was 36%. CPV-2 infection were associated with age, breed, sampling season and immunization status, but not with gender, publication time and diagnostic methods.

**Conclusions:**

Our results indicated that CPV-2 is prevalent among dogs in China. It is therefore necessary to carry out continuous surveillance and epidemiological studies of CPV-2. In addition, accordingly, effective measures should be taken to prevent the transmission and spread of CPV-2 among the Chinese dog population.

## Background

Canine parvovirus 2 (CPV-2) is a linear, non-segmented, single-stranded DNA virus that belongs to the family *Parvoviridae* and causes a highly infectious disease [1]. The main clinical characteristics of CPV-2 infection are acute gastroenteritis symptoms, such as vomiting, fever, leucopoenia, and diarrhoea that affect dogs of different ages, especially for young puppies 6 months and younger [2]. CPV-2 infection is usually acquired through contact with infected dog faeces, vomit, saliva, and contaminated water or food. It was reported that the prevalence of CPV-2 was correlated with age, season, immune status and regional distribution [3]. In addition, the prevalence of CPV-2 also showed seasonal characteristics. Generally speaking, the infection is more serious in the spring, late autumn and early winter [4].

CPV-2 is a potentially fatal pathogen in domestic dogs and other canine species. It may also infect other animals, such as cats because it has evolved into variant types that can infect cats [5]. Studies have shown that CPV-2 is a variant of the feline parvovirus (FPV)-like virus that was found in faecal samples from dogs with diarrhoea and quickly spread around the world [6]. Subsequently, the CPV-2, which had previously been unable to infect cats, has been replaced by different but closely related antigen CPV-2 variants and is capable of infecting cats, suggesting that CPV-2 may have the ability to spread across species [7]. A transformation of animal virus into a zoonotic virus, either by mutation or by recombination, has been reported. Examples of host switching viruses include the severe acute respiratory syndrome coronavirus (SARS-CoV) [8], Middle East respiratory coronavirus (MERS-CoV) [9], and some subtypes of influenza A virus (IAV) [10–12]. Therefore, the analysis of animal virus infection rates and epidemiological characteristics is necessary to reduce the risk of cross-species transmission between animals and humans and to prevent the potential threat of animal virus pandemic among humans.

CPV-2 was first reported in the USA in 1978 and has become prevalent worldwide, especially in China and other Asian countries [13, 14]. In 1978, there was a large outbreak of mixed infection of CPV-2 and canine coronavirus (CCoV) in dogs in the USA with high morbidity and mortality, attracting extensive global attention [15]. In China, the first record of CPV-2 was in 1982, and the infections were reported in widespread regions of China because of the high morbidity and mortality [16]. Since then, a number of studies on CPV-2 infection have been performed in China. Currently, CPV-2 infection has previously been reported in 23 provinces in China, and a long-term investigation has revealed that the rates of CPV-2 infection among Chinese domestic dog populations varied from 5.9 to 85.9% [17, 18]. These data provide a basic reference for our understanding of the epidemiological characteristics of CPV-2 in China. However, regional epidemiological studies are limited by sample size, sampling location, and season because China is a large country with a diverse climate. Therefore, the prevalence and risk factors of CPV-2 in China are not fully understood. Hence, this study focussed on a systematic review and meta-analysis to summarise the prevalence of CPV-2 and examine the potential risk factors of CPV-2 infection in mainland China.

## Materials and methods

### Search strategy

The study search was planned and performed according to the Meta-analysis of Observational Studies in Epidemiology guidelines [19]. To identify the epidemiological studies on CPV-2 in China, the literature published either in English or Chinese was searched up to December 2019. English databases (PubMed, Google Scholar, Cochrane library, and Clinical Trials) and Chinese databases (CNKI, Cqvip, WANFANG data, and Baidu scholar) were searched using “Canine parvovirus or CPV”, “epidemiology or incidence or prevalence”, “dog or canine”, and “China or Chinese”, or variants and combinations of these words, as keywords. Studies included in this systematic review had to contain any epidemiological data related to CPV-2 among dog populations from mainland China.

### Exclusion criteria

The following studies were excluded from this systematic review and meta-analysis: (1) data from countries and regions outside mainland China; (2) literature that had review studies, case reports, press releases, newsletters, forums, and questionnaire surveys; (3) non-epidemiological studies (e.g., basic research); (4) no clear sampling time, sample size, infection rate, and prevalence rate in the study; and (5) insufficient information and duplicated findings.

### Data extraction

The corresponding data were extracted from studies that met inclusion criteria and extracted into a Microsoft Excel datasheet. Recorded bibliographic data contained the following information: province, study design, background information, sample size, detection method, publication year, author, detection method, and sampling season.

### Quality of publications

The selected publications were independently evaluated by two reviewers based on the established inclusion criteria. These publications were selected based on the information provided in the title and/or abstract as well as their full-text availability in Chinese or English. Furthermore, these publications had to contain prevalence data related to CPV-2 in mainland China. The quality of selected publications was assessed using the Newcastle–Ottawa scale (NOS). Studies with scores of 5 or above (out of 10) were included in the meta-analysis.

### Statistical analysis

This study was planned and performed according to the Preferred Reporting Items for Systematic Reviews and Meta-Analysis (PRISMA) [20]. In the eligible studies, a random effects model was utilised to calculate the pooled prevalence of CPV-2 infection among domestic dogs. Meta-analysis was performed using Review Manager 5.3 (Copenhagen: The Nordic Cochrane Centre, The Cochrane Collaboration, 2014). The pooled estimates were the outcome of the meta-analysis and visualised the heterogeneity among the included studies using Forest plots. Forest plots were used to summarise estimates with 95% confidence intervals (CIs). The heterogeneity index among the included studies was determined using the Cochrane’s Q test (chi-squared) and Higgins I^2^ statistics. An I^2^ > 50% represents substantial heterogeneity [21]. Potential publication bias was assessed using a funnel plot. It was considered significant when the *P* value was less than 0.05.

## Results

### Description of studies

**Fig. 1:**
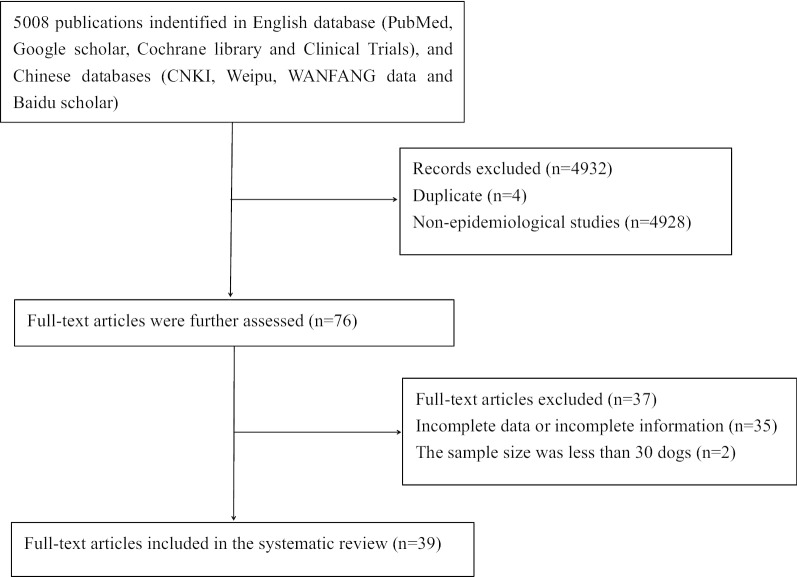
Flow chart for screening eligible studies

Based on search strategies of databases, a total of 5008 Chinese or English articles were identified. After the preliminary screen, 76 full-text articles were selected, and papers, duplicate citations, and studies not relevant to the current meta-analysis were removed. After excluding 37 articles with incomplete data, 39 articles met the inclusion criteria and were included in the systematic review (Fig. 1). The articles were published between 2006 and 2019, and covered 20 provinces in China. A cross-sectional study of all articles was performed, and period prevalence was calculated (Table 1).

**Table 1:** Included studies of CPV-2 infection among dogs in mainland China

Reference	Publication year	Province	No. examined	No. positive	Diagnostic methods	Study design
Zhao [17]	2016	Henan	19,907	1169	PCR	Cross sectional
Wu [22]	2014	Beijing	352	55	PCR	Cross sectional
Wang [8]	2016	Beijing	58	43	Antigen Rapid CPV Ag kit	Cross sectional
Zhao [7]	2017	Heilongjiang	216	152	PCR	Cross sectional
Li [23]	2010	Guangxi	45,199	3174	unknown	Cross sectional
Zhang [24]	2011	Beijing	404	115	Antigen Rapid CPV Ag kit	Cross sectional
Bai [25]	2011	Beijing	327	51	PCR	Cross sectional
Zhang [26]	2016	Beijing	1209	232	PCR	Cross sectional
Zhao [18]	2013	Beijing	269	231	Antigen Rapid CPV Ag kit	Cross sectional
Chen [27]	2019	Sichuan	145	120	PCR	Cross sectional
Fu [28]	2017	Jilin	339	154	Antigen Rapid CPV Ag kit	Cross sectional
Zhang [29]	2019	Jilin	526	176	PCR	Cross sectional
Zhuo [30]	2015	Jiangsu	4290	704	Antigen Rapid CPV Ag kit	Cross sectional
Jing [31]	2018	Gansu	4052	1028	Antigen Rapid CPV Ag kit	Cross sectional
Zhang [32]	2013	Henan	495	88	unknown	Cross sectional
Lou [33]	2010	Jiangsu	2319	711	Antigen Rapid CPV Ag kit	Cross sectional
Tai [34]	2008	Jiangsu	2824	191	Antigen Rapid CPV Ag kit	Cross sectional
Huang [35]	2018	Guangxi	683	378	Antigen Rapid CPV Ag kit	Cross sectional
Kang [36]	2016	Shandong	2682	296	Antigen Rapid CPV Ag kit	Cross sectional
Yang [37]	2006	Sichuan	2493	1873	Antigen Rapid CPV Ag kit	Cross sectional
Yang [38]	2012	Chongqing	300	60	Unknown	Cross sectional
Geng [39]	2009	Beijing	189	137	Antigen Rapid CPV Ag kit	Cross sectional
Fu [40]	2012	Jilin	5684	4124	Antigen Rapid CPV Ag kit	Cross sectional
Yang [41]	2014	Jilin	1864	1410	Antigen Rapid CPV Ag kit	Cross sectional
Zeng [42]	2013	Shanxi	6027	956	Antigen Rapid CPV Ag kit	Cross sectional
Zhao [43]	2013	Ningxia	1085	360	Antigen Rapid CPV Ag kit	Cross sectional
Lin [44]	2011	Liaoning	96	78	PCR	Cross sectional
Sun [45]	2016	Shandong	1511	295	Antigen Rapid CPV Ag kit	Cross sectional
Ju [46]	2012	Shanghai	338	86	PCR	Cross sectional
Chen [47]	2012	Zhejiang	4613	644	Unknown	Cross sectional
Zan [48]	2017	Tianjin	182	35	Unknown	Cross sectional
Luo [49]	2014	Zhejing	578	113	Antigen Rapid CPV Ag kit	Cross sectional
Ma [50]	2014	Inner Mongolia	243	134	Antigen Rapid CPV Ag kit	Cross sectional
Zhao [51]	2014	Hubei	8577	1517	Antigen Rapid CPV Ag kit	Cross sectional
Chen [52]	2016	Sichuan	1360	203	Antigen Rapid CPV Ag kit	Cross sectional
Han [53]	2014	Xinjiang	130,40	4925	Antigen Rapid CPV Ag kit	Cross sectional
Wu [54]	2011	Xinjiang	2582	863	Antigen Rapid CPV Ag kit	Cross sectional
Ye [55]	2016	Hunan	362	268	Antigen Rapid CPV Ag kit	Cross sectional
Wu [56]	2012	Henan	427	315	Antigen Rapid CPV Ag kit	Cross sectional

Based on search strategies of databases, a total of 39 articles met the inclusion criteria and were included in the systematic review. The included studies, published between 2006 and 2019, covered 20 provinces in China. A cross-sectional studies were carried out in all included studies. Data information such as publication year, sampling location, sample size, positive number, diagnostic method and study design were extracted from all included studies

### Prevalence of CPV-2 infection in mainland China

**Fig. 2:**
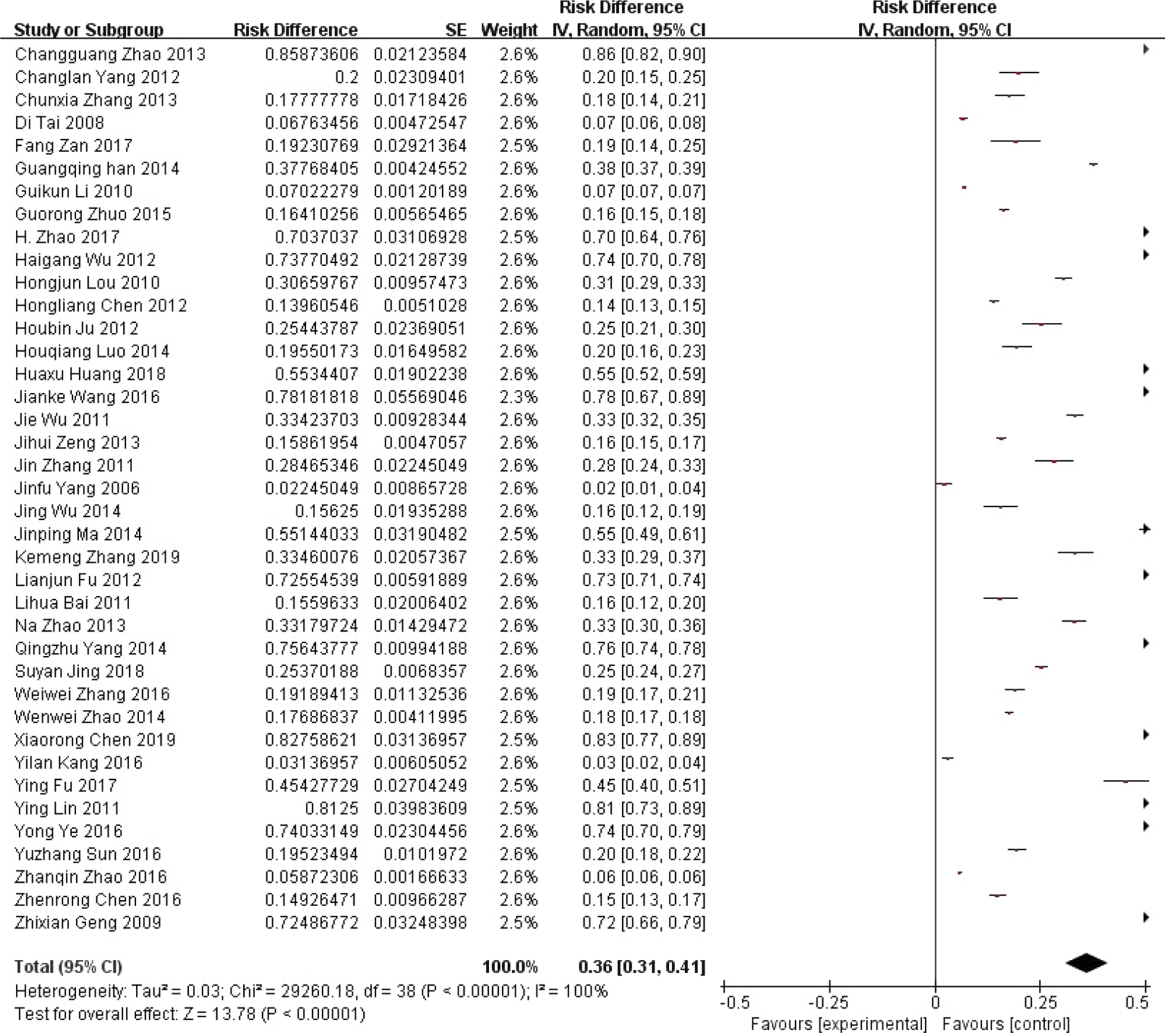
Random-effects meta-analysis of CPV-2 infection in mainland China

**Fig. 3:**
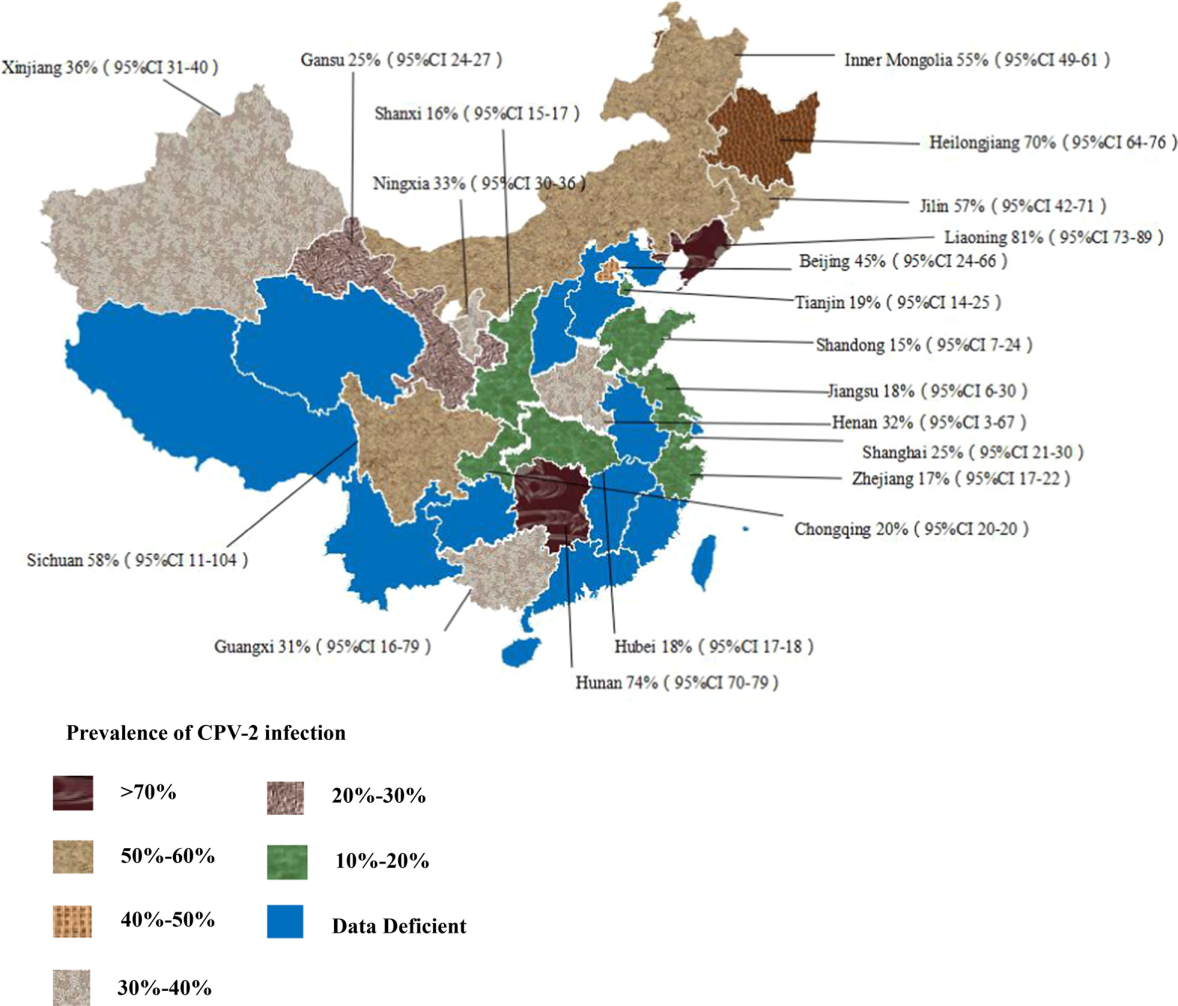
Map of CPV-2 infection in mainland China. Northeast China: Heilongjiang, Jilin, Liaoning; Northern China: Inner Mongolia, Shanxi, Hebei, Beijing, Tianjin; Northwest China: Xinjiang, Qinghai, Gansu, Ningxia, Shaanxi; Eastern China: Shandong, Anhui, Jiangxi, Jiangsu, Zhejiang, Shanghai, Fujian; Southern China: Guangxi, Guangdong, Shenzhen, Hainan, Macao, Hong Kong; Central China: Henan, Hunan, Hubei; Southwest China: Tibet, Yunnan, Guizhou, Sichuan, Chongqing

A total of 137,844 domestic dogs and 27,464 CPV-2-positive cases were included in the meta-analysis. The total prevalence of CPV-2 in mainland China was 36% at 95% CI (0.31, 0.41), and demonstrated a strong heterogeneity (Chi^2^ = 29,260.2, I^2^ = 100%, *P* < 0.00001) (Fig. 2). Data from 39 studies were collected from 20 provinces, with the eastern and northern provinces being the majority. Among those provinces, the prevalence in Liaoning and Hunan provinces were higher than 70%, while that in most provinces of northern China reached above 30% (Fig. 3). The prevalence of CPV-2 in administrative districts of China (from highest to lowest) was as follows: 63% in Northeast China, 48% in Southwest China, 43% in North China, 38% in Central China, 29% in Northwest China, and 18% in East China. The prevalence of CPV-2 in Northeast China was higher than that in other administrative districts of China (Table 2).

**Table 2:** Infection of CPV-2 in dogs in mainland China

	No. studies	No. positive	No. test	% (95% CI)	Chi^2^	P	I^2^ (%)
Region							
Northeast China	6	6094	8725	63% (52–74)	465.1	0	99
North China	9	1033	3230	43% (25–61)	1027.7	0	99
Northwest China	5	8132	26,786	29% (19–39)	1256.3	0	100
East China	8	3040	19,155	18% (13–23)	641.6	0	99
Central China	5	3357	29,768	38% (24–51)	2521.2	0	100
Southwest China	4	2256	4298	48% (10–86)	2423.1	0	100
Age							
Under 6 months of age	25	14,659	30,657	68% (52–85)	33,254.4	0	100
6 months of age or above	25	5372	28,386	20% (13–26)	6666.9	0	100
Immunization status							
Yes	24	5123	20,346	20% (15–26)	3172.5	0	99
No	24	14,434	20,679	68% (62–75)	3152.9	0	99
Gender							
Male	13	5235	20,647	45% (30–61)	6961.5	0	100
Female	13	4194	18,323	38% (25–50)	4175.6	0	100
Breed							
Purebred	15	9899	16,954	66% (50–82)	10,310.2	0	100
Mutt	15	4262	14,186	24% (16–33)	2682.9	0	99
Published time							
Before 2016	25	22,915	104,615	35% (27–43)	24,380.0	0	100
2016 or later	14	4549	32,867	40% (30–49)	3432.4	0	100
Season							
Spring	20	4684	20,887	34% (27–41)	3357.8	0	99
Summer	18	2522	19,769	15% (11–18)	1151.1	0	98
August	18	3382	18,894	23% (18–28)	1642.8	0	99
Winter	18	3839	18,667	24% (18–30)	2413.3	0	99
Diagnostic methods							
Antigen Rapid CPV Ag Test kit	25	21,344	63,939	43% (34–52)	18,492.7	0	100
PCR	9	2119	23,116	39% (21–53)	1772.5	0	100
Unknown	5	4001	50,789	15% (10–20)	257.3	0	98

The corresponding data related to the prevalence of CPV-2 infection in dogs were analyzed, including administrative region, age, immunization status, gender, breed, sampling season and diagnostic methods. Among these risk factors, the occurrence of CPV-2 infection was significantly associated with age, breed, sampling season and immunization status, but not with gender and diagnostic methods in dogs. Northeast China: Heilongjiang, Jilin, Liaoning; Northern China: Inner Mongolia, Shanxi, Hebei, Beijing, Tianjin; Northwest China: Xinjiang, Qinghai, Gansu, Ningxia, Shaanxi; Eastern China: Shandong, Anhui, Jiangxi, Jiangsu, Zhejiang, Shanghai, Fujian; Southern China: Guangxi, Guangdong, Shenzhen, Hainan, Macao, Hong Kong; Central China: Henan, Hunan, Hubei; Southwest China: Tibet, Yunnan, Guizhou, Sichuan, Chongqing. CI, confidence interval; Chi^2^, chi-square; *P*, *P* value

### Correlates of CPV-2 prevalence

As shown in Table 2, we analysed the risk factors related to the prevalence of CPV-2 infection in dogs. Among these risk factors, the occurrence of CPV-2 infection was significantly associated with age, sampling season, immunisation status, and breed: (1) the prevalence of infection in young dogs under 6 months of age was 68%, while that in dogs 6 months of age or above was 20%, which is a significant difference (*P* < 0.05); (2) the prevalence in unimmunised dogs (68%) was higher than that in immunised dogs (20%), and shows a significant difference (*P* < 0.05); (3) the prevalence of CPV-2 was the highest in spring (34%) and the lowest in summer (15%), which was significantly different (*P* < 0.05). The infection was more frequent in spring than in other seasons; (4) the prevalence of infection in purebred dogs was 66%, while in mutts it was 24% (*P* < 0.05), indicating that CPV-2 was more susceptible in purebred dogs than in mutts. However, the prevalence of CPV-2 in male dogs was 45%, and in female it was 38%; The prevalence based on the antigen Rapid CPV Ag Test kit was 43%, and based on the PCR it was 39%; The prevalence relying to publication year of included studies was as follows: Before 2016 35% and 2016 or later 40%. These results all shows no significant difference (*P* > 0.05), indicating that CPV-2 prevalence was less affected by gender, diagnostic methods and published time.

## Discussion

To the best of our knowledge, the present study is the first meta-analysis investigating the prevalence of CPV-2 infection in domestic dogs in mainland China. In recent years, a large number of studies on CPV-2 have provided a deeper understanding of CPV-2 infection in Chinese domestic dogs. Statistics of the infection rates and epidemic characteristics of CPV-2 in the region are available through epidemiological studies; however, a large sample size is required to reduce the sampling error. This is because as the number of samples increases, the sample gets closer to the population. Furthermore, the climate in the north and south of China are different, which may have an impact on the prevalence of CPV-2. To understand the prevalence and epidemiological characteristics of CPV-2 in China, it is not possible to simply integrate the epidemiological data collected at different times and locations. Therefore, this study focussed on a systematic review and meta-analysis to summarise the prevalence of CPV-2 and examine the potential risk factors of CPV-2 infection in mainland China.

The estimates provided in our studies were based on data from 20 provinces in mainland China, and it demonstrated that the total prevalence of CPV-2 in mainland China was 36%. Statistics of subgroups showed that the prevalence of CPV-2 in northeast China was 63%, which was significantly higher than that in other administrative districts. Moreover, the highest rate (81%) of prevalence is observed in Liaoning province than in other provinces. The prevalence of CPV-2 varied from 15 to 81% in 20 provinces. Therefore, our study shows that CPV-2 is prevalent in Chinese dogs.

Although dogs of all ages can be infected with CPV-2, puppies are more susceptible, and become infected by CPV-2 show illness within 3–7 days, presenting with severe gastroenteritis, lethargy, vomiting, fever, and diarrhoea [57–59]. In this study, we observed that the prevalence of CPV-2 infection was significantly higher in puppies under 6 months of age, confirming that puppies were at a greater risk of contracting CPV-2 compared to adult dogs. This difference might be due to immature development of immune organs and lymphoid tissues, resulting in weakened body resistance in puppies [60]. Moreover, the prevalence of CPV-2 in purebred dogs was lower than that in hybrids in this study. These differences may be attributed to hybrids being able to adapt better to local climates and conditions, and developing more resistance to CPV-2 [37].

According to the current subgroup meta-analysis, the prevalence of CPV-2 was 34% in spring, which was higher than in other seasons. Spring is characterized by greater temperature differences between day and night, and if the dogs' immune system do not adapt to these temperature differences, that can decrease dogs’ immunity, which could be the reason for the seasonal variations in the prevalence of CPV-2 infection. In addition, the lower critical temperature for CPV-2 survival may also explain the seasonal variation in CPV-2 infection rates. Furthermore, several studies have reported that CPV-2 infection rates were higher in spring, which could be because people spend more time walking their dogs outdoors in the spring, thus increasing the chances of dogs being exposed to viral pathogens in the environment [17]. Therefore, these results showed that CPV-2 infection occurs throughout the year and is more prevalent in the spring. It is suggested that dogs be kept warm when the temperature between day and night varies greatly in spring. In addition, outdoor activity should be reduced to decrease the risk of CPV-2 infection.

Analyzing the subgroups, the prevalence of CPV-2 in unvaccinated dogs was significantly higher than those in vaccinated dogs. The reason for this is that dogs injected with vaccine can resist infection as they produce high levels of antibodies. However, interestingly, there was also prevalence (20%) in immunised dogs in this study. This could be attributed to improper immunisation procedures, improper preservation of vaccine, and inaccurate vaccination dose leading to immune failure. It could be because some Chinese dog owners prefer to get their dogs vaccinated at a kennel or pet shop rather than at an animal hospital. In addition, some dog owners are reluctant to cooperate with the hospital to have their dogs fully examined, which results in the immune effect not being detected effectively [29]. Therefore, enhancing the scientific awareness of dog owners, standardising immunisation procedures, and strengthening supervision over the transportation and preservation of vaccines are keys to improving immunisation efficiency.

The application of in-clinic immunochromatographic assays is available for the diagnosis of CPV infection in everyday veterinary practise, as the procedure is simple, inexpensive, and timely [61]. It only requires a faecal sample to permit diagnosis in vivo, which can assist in the early diagnosis of CPV. Meanwhile, PCR technology is also used for the investigation of CPV-2, as it is rapid, efficient, and highly accurate [62]. In the current study, the prevalence found in PCR was slightly lower than that found in in-clinic immunographic assays. This could be due to the high sensitivity and specificity of PCR to identify the species level and its acceptable genetic diversity. However, the difference in prevalence detected by the two diagnostic methods was not significant (*P* > 0.05), indicating that there is a fair agreement between in-clinic immunographic assay and PCR findings [63].

Our study had several limitations. First, one study identified during our systematic review did not have full text, leading to loss of qualified data. Second, the factors available for analyses were limited, with only publication date, geographical location, sampling season, gender, breed, diagnostic methods, and immunisation status retained. As a result, other potential risk factors were not analysed. Furthermore, the 39 included studies were cross-sectional studies. Therefore, more high-quality epidemiological studies on CPV-2 infection in Chinese domestic dogs should be carried out in the future to gain a more comprehensive understanding of the current situation of CPV-2 in China.

## Conclusions

In conclusion, based on the results of this study, we found that the CPV-2 is prevalent in mainland China and even highly prevalent in some regions in China. In addition, the results illustrated correlation between CPV-2 prevalence and seasonality, a dog’s age/gender/breed/vaccination. Furthermore, the results suggest there is a need for continuous research on CPV-2 infection in more dogs to help other researchers to delve into the risk factors for CPV-2 infection, and indicate effective measures should be taken to reduce the prevalence according to the risk factors for CPV-2 infection.

## Data Availability

The data analyzed during the current study was available from the corresponding author on reasonable request.

## Abbreviations

CPV-2: Canine parvovirus 2
CCoV: Canine coronavirus
SARS-CoV: Severe acute respiratory syndrome coronavirus
MERS-CoV: Middle East respiratory coronavirus
IAV: Influenza A virus
MOOSE: Meta-analysis of observational studies in epidemiology
NOS: Newcastle–Ottawa scale
PRISMA: Preferred reporting items for systematic reviews and meta-analysis
PCR: Polymerase chain reaction
